# Prior upregulation of interferon pathways in the nasopharynx impacts viral shedding following live attenuated influenza vaccine challenge in children

**DOI:** 10.1016/j.xcrm.2021.100465

**Published:** 2021-12-09

**Authors:** André G. Costa-Martins, Karim Mane, Benjamin B. Lindsey, Rodrigo L.T. Ogava, Ícaro Castro, Ya Jankey Jagne, Hadijatou J. Sallah, Edwin P. Armitage, Sheikh Jarju, Bankole Ahadzie, Rebecca Ellis-Watson, John S. Tregoning, Colin D. Bingle, Debby Bogaert, Ed Clarke, Jose Ordovas-Montanes, David Jeffries, Beate Kampmann, Helder I. Nakaya, Thushan I. de Silva

**Affiliations:** 1 Department of Clinical and Toxicological Analyses, School of Pharmaceutical Sciences, University of São Paulo, São Paulo, Brazil; 2 Medical Research Council Unit The Gambia at the London School of Hygiene and Tropical Medicine, P.O. Box 273, Fajara, The Gambia; 3 The Florey Institute for Host-Pathogen Interactions and Department of Infection, Immunity and Cardiovascular Disease, The University of Sheffield, Sheffield S10 2RX, UK; 4 The University of Edinburgh/MRC Centre for Inflammation Research, The Queen’s Medical Research Institute, Edinburgh EH16 4TJ, UK; 5 Department of Infectious Disease, Imperial College London, London W2 1NY, UK; 6 Division of Gastroenterology, Hepatology, and Nutrition, Boston Children’s Hospital, Boston, MA 02115, USA; 7 Program in Immunology, Harvard Medical School, Boston, MA 02115, USA; 8 Broad Institute of MIT and Harvard, Cambridge, MA 02142, USA; 9 Harvard Stem Cell Institute, Cambridge, MA 02138, USA; 10 The Vaccine Centre, London School of Hygiene and Tropical Medicine, Keppel Street, London WC1E 7HT, UK; 11 Scientific Platform Pasteur, University of São Paulo, São Paulo, Brazil

**Keywords:** LAIV, influenza, interferon-stimulated genes, mucosal, transcriptome, asymptomatic respiratory viral infection

## Abstract

In children lacking influenza-specific adaptive immunity, upper respiratory tract innate immune responses may influence viral replication and disease outcome. We use trivalent live attenuated influenza vaccine (LAIV) as a surrogate challenge model in children aged 24–59 months to identify pre-infection mucosal transcriptomic signatures associated with subsequent viral shedding. Upregulation of interferon signaling pathways prior to LAIV is significantly associated with lower strain-specific viral loads (VLs) at days 2 and 7. Several interferon-stimulated genes are differentially expressed in children with pre-LAIV asymptomatic respiratory viral infections and negatively correlated with LAIV VLs. Upregulation of genes enriched in macrophages, neutrophils, and eosinophils is associated with lower VLs and found more commonly in children with asymptomatic viral infections. Variability in pre-infection mucosal interferon gene expression in children may impact the course of subsequent influenza infections. This variability may be due to frequent respiratory viral infections, demonstrating the potential importance of mucosal virus-virus interactions in children.

## Highlights

• Upregulated mucosal interferon prior to vaccination leads to reduced LAIV shedding
• Asymptomatic respiratory viral infections were seen in 42% of children
• Other respiratory viral infections contribute to higher interferon gene expression

## Graphical abstract

**Figure:**
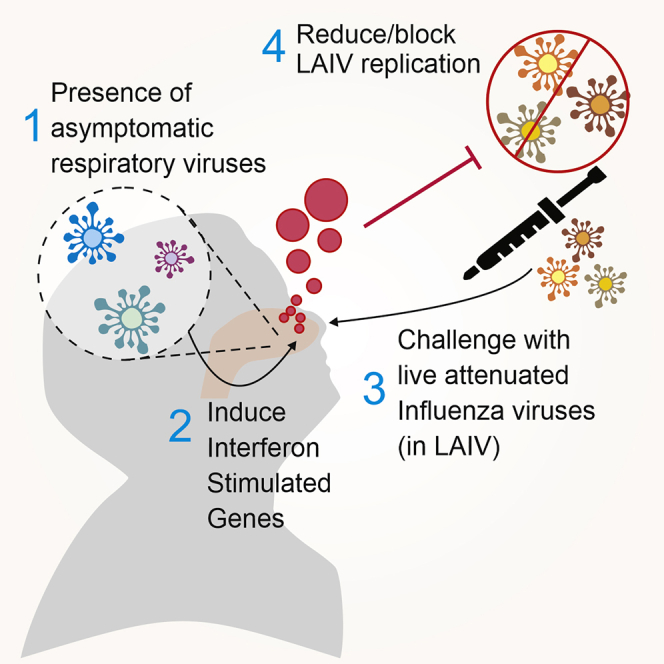

## Teaser

Costa-Martins et al. show that in children seronegative to influenza vaccine strains, upregulated nasopharyngeal interferon genes prior to LAIV receipt associates with reduced vaccine strain shedding. Asymptomatic respiratory viral infections are common and result in higher interferon gene expression, highlighting the importance of virus-virus interactions in the upper respiratory tract.

## Introduction

Influenza results in significant pediatric morbidity and mortality worldwide, with the largest burden in low- and middle-income countries.^1^ Historical human challenge studies defined the main known correlate of protection from influenza in adults based on serum hemagglutination inhibition (HAI) titers.^2^ In children who are influenza naive and have no pre-existing adaptive immune responses, innate immunity in the upper respiratory tract (URT) mucosa may be important in determining the risks of infection as well as severity of disease.

Unlike in adults, ethical and feasibility constraints of conducting influenza challenge studies in children limit the ability to use this controlled experimental approach to gain insight into such aspects of protective immunity. We hypothesized that live attenuated influenza vaccines (LAIVs) could be used as a surrogate challenge model in children, with shedding of vaccine strains as the primary endpoint of interest. LAIVs contain influenza strains that replicate in the cooler URT only, showing induction of interferon-stimulated genes (ISGs) and replication kinetics similar to that of wild-type strains.^3^^,^^4^ Millions of children worldwide ≥2 years of age have received LAIVs for protection against seasonal influenza viruses to date, with an excellent safety profile.

Using LAIV challenge in children with no detectable serum HAI titers to vaccine strains, we sought to define pre-challenge transcriptome signatures in the nasopharyngeal mucosa that were associated with the degree of viral shedding several days later; thus, mimicking what may determine the outcome of URT influenza infections in influenza-naive children. We show that many children have upregulated interferon pathways at the time of vaccination that correlate with limited influenza strain shedding and that upregulated ISG signatures may, in part, be explained by asymptomatic infection with respiratory viruses such as human rhinoviruses.

## Results

### Study participants

**Figure 1:**
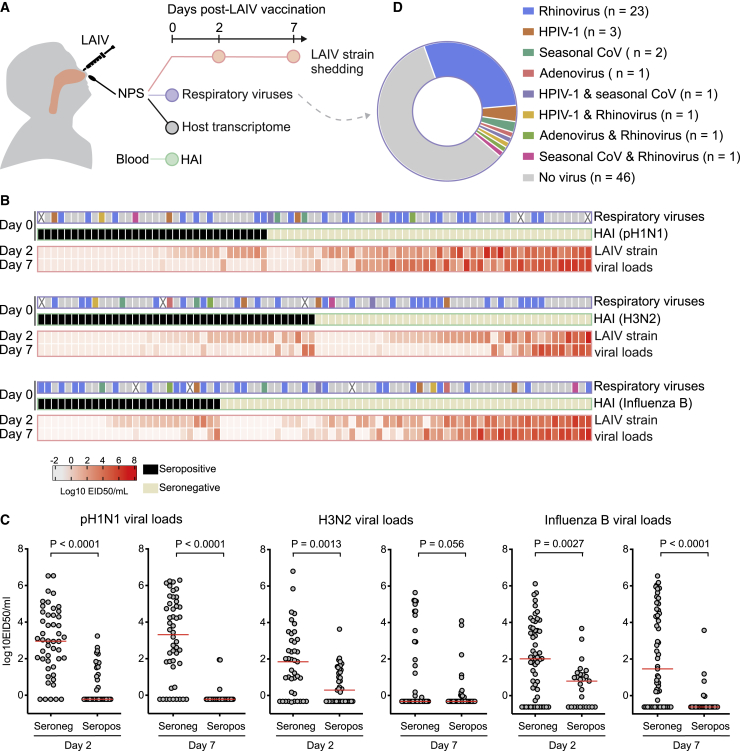
Study design, nasopharyngeal LAIV viral loads, and presence of asymptomatic respiratory viruses (A) Study design and sampling. Influenza vaccine naive children (n = 82) given a single dose of the northern hemisphere 2017-18 LAIV (Nasovac-S Serum Institute of India) were included in the study. NPS, nasopharyngeal swab; HAI, hemagglutinin inhibition titer. (B) Heatmap showing nasopharyngeal viral shedding at days 2 and 7 following LAIV (log_10_ 50% egg infectious dose equivalent [EID_50_]/mL) for 2009 pandemic H1N1 (pH1N1), H3N2, and influenza B viruses in children seronegative (HAI titer < 1:10) and seropositive for each corresponding strain. Detection of asymptomatic respiratory viruses at baseline are displayed, with key for each virus as per Figure 1D (X indicates samples not available for testing). HPIV-1, human parainfluenza 1; seasonal CoVs, seasonal coronaviruses (229E, OC43, NL63). X denotes children where no result was available due to lack of sample availability. (C) Comparison of day 2 and day 7 nasopharyngeal viral loads in children who are seronegative and seropositive to each influenza strain. Red lines denote median value. The p values are from Mann-Whitney U test. (D) Prevalence of asymptomatic respiratory virus in 33/79 (41.8%) children in nasopharyngeal swabs taken prior to vaccination.

Influenza vaccine-naive children aged 24–59 months were given one dose of trivalent LAIV (Nasovac-S, Serum Institute of India) as part of a previously described open-label, prospective, observational, phase 4 immunogenicity study in The Gambia (ClinicalTrials.gov NCT02972957).^5^ Children eligible for the study were clinically well, with no history of respiratory illness within the last 14 days. We have previously demonstrated the role of serum antibody in reducing LAIV shedding in children, with no effect seen from influenza-specific mucosal immunoglobulin A (IgA) or T cell responses.^5^ To evaluate mucosal factors influencing LAIV strain shedding in influenza-naive children, in the current study we included children who were seronegative (HAI titer < 1:10) to at least one strain and from whom we had adequate nasopharyngeal RNA for transcriptome profiling (n = 82). Of these, 48 were seronegative to pH1N1, 41 were seronegative to H3N2, and 55 were seronegative to influenza B. The study design is detailed in Figure 1A.

### LAIV viral loads and presence of asymptomatic respiratory viruses at baseline

Nasopharyngeal viral loads at both day 2 and day 7 were significantly lower in seropositive than seronegative children for a given influenza strain (Figures 1B and 1C). However, a wide range was seen within the seronegative children, with some viral loads equivalent to those in seropositive children, whereas others were very high at both day 2 and day 7 (Figure 1C).

Asymptomatic respiratory viral infections at baseline were detected in 41.8% (33/79) of children with valid multiplex RT-PCR assay results (Figure 1D). Twenty-six had rhinovirus (three of these with other viral co-infections), five had human parainfluenza virus-1 (HPIV-1; two with co-infections), four had seasonal coronavirus (CoV) (two with co-infections), and two had adenovirus (one with co-infection). Asymptomatic respiratory viruses were detected in 20/39 children aged 24–35 months, compared with 9/30 children aged 36–47 months and 4/10 children aged 48–59 months. Of note, the study was conducted during the dry season in The Gambia, when circulation of respiratory viruses is generally lower than during the wet season.^6^

### Transcriptional profiles associated with LAIV shedding and presence of asymptomatic respiratory viruses

**Figure 2:**
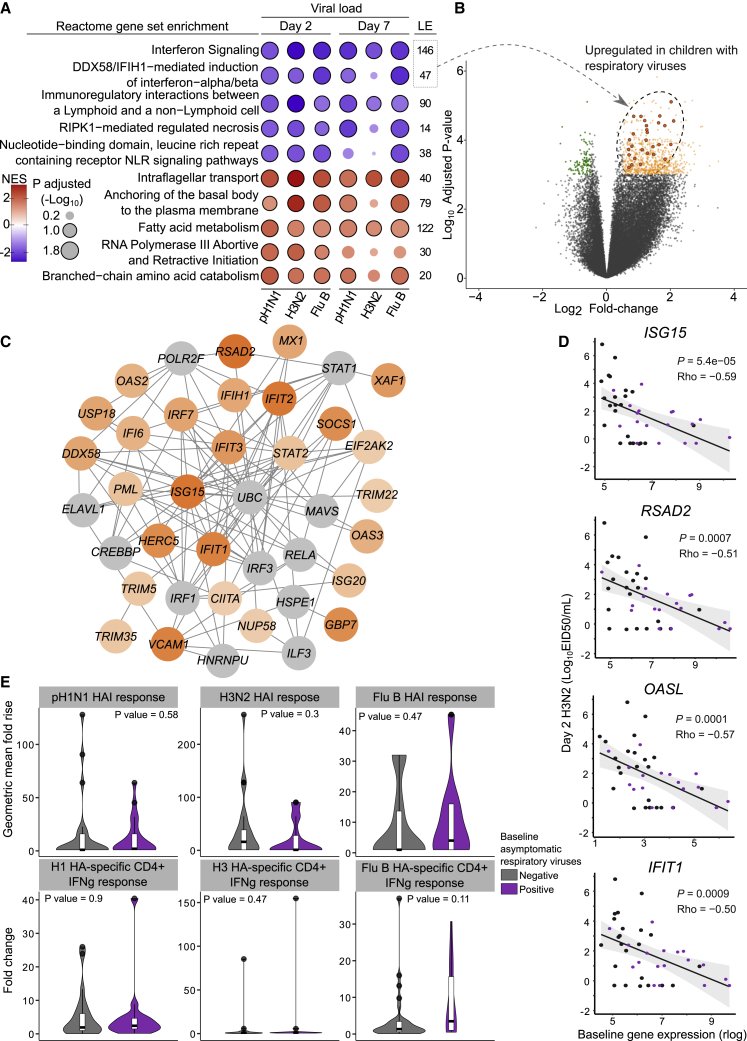
Baseline transcriptional profiles associated with LAIV strain shedding at day 2 and day 7 in children seronegative to each influenza strain prior to vaccination (A) Selected pathways from gene set enrichment analysis (GSEA) using the Spearman correlation coefficients between normalized gene expression (rlog) at baseline and strain-specific viral loads (log_10_ EID_50_/mL) at day 2 and day 7 as rank, and Reactome pathways set. NES, normalized enrichment score. The size of circles is proportional to the adjusted −log_10_ p value from GSEA, while the intensity of the circle color denotes the NES for each pathway/strain-specific viral load combination (deeper color indicates higher NES). Blue circles denote enrichment of genes in nasopharynx prior to LAIV challenge that negatively correlate with viral load at day 2 and day 7, and red circles denote enrichment of genes that positively correlate with viral loads. Significant pathways (adjusted p value < 0.1) are highlighted with a black outline. The number of leading edge (LE) genes is provided for each pathway, i.e., genes contributing to the enrichment signal. (B) Volcano plot of differentially expressed genes (DEGs) at baseline in children with (n = 33) and without (n = 46) asymptomatic viral infections, defined by a log_2_ fold-change of ±0.5 and adjusted p value of 0.001. The 28 genes from interferon (IFN) signaling pathways in (A) also found in upregulated DEGs are highlighted. (C) Protein-protein interaction network of overlapping genes (n = 27, shown in orange) from IFN signaling pathways shown in (A) and upregulated DEGs in children with asymptomatic respiratory viruses shown in (B). Network was constructed using Network Analyst,^7^ the InnateDB interaction database, and the minimum network option. Proteins in orange scale are colored by log_2_ fold-change of corresponding gene in the DEG analysis. Additional connecting protein nodes not found as DEGs, but are key within the network are shown in gray. Of these, CREBBP, IRF1, RELA, STAT1, and UBC are found within the IFN signaling pathway LE genes. (D) Examples of inverse correlation between baseline IFN gene expression and LAIV strain viral load (day 2, H3N2). Rho represents Spearman correlation coefficient. Values from children with asymptomatic respiratory viruses detected at baseline are colored purple. (E) Antibody response (HAI) and hemagglutinin (HA)-specific CD4+ T cell response to LAIV strains in children with and without evidence of asymptomatic respiratory viral infections at baseline. Antibody response is expressed as the fold-rise in geometric mean titer after vaccination and CD4+ T cell response as the fold-change after vaccination in the percentage of CD4+ T cells expressing IFN gamma (IFNg) following stimulation with strain-specific HA peptides. The p values are from Mann-Whitney U test. Shown are median and interquartile range for each plot.

Gene set enrichment analysis (GSEA) was conducted to identify mucosal transcriptional profiles from nasopharyngeal swabs taken prior to LAIV challenge that may influence replication of LAIV strains. Correlation coefficients were generated between mucosal gene expression at baseline and viral loads at days 2 and 7 for each strain. Negative normalized enrichment scores (NESs) from the GSEA indicate pathways enriched for genes whose expression inversely correlates with viral loads and vice versa. Five pathways were significantly enriched (adjusted p value < 0.1) for genes associated with lower viral loads for all three strains at day 2, and of these five pathways, two were also significant for all three strains at day 7 (Interferon signaling and Immunoregulatory interactions between a lymphoid and non-lymphoid cell; Figure 2A; see Table S1 for NESs and adjusted p values). Greater expression of genes at baseline enriched in the Intraflagellar transport and Fatty acid metabolism pathways were associated with higher viral loads at both day 2 and day 7.

To see whether the presence of asymptomatic respiratory viral infection could explain upregulated genes from interferon signaling pathways in some children, differentially expressed genes (DEGs) between children with and without viral infections at baseline were identified (817 upregulated, 114 downregulated; Table S2). Twenty eight significantly upregulated DEGs (Figure 2B) were also found within the gene set contributing to the GSEA enrichment signal in interferon signaling pathways (leading edge genes) shown in Figure 2A. The expression of these genes at baseline was among those negatively correlated with LAIV viral loads at days 2 and 7 (*CIITA*, *DDX58*, *EIF2AK2*, *GBP4*, *GBP7*, *HERC5*, *IFI6*, *IFIH1*, *IFIT1*, *IFIT2*, *IFIT3*, *IRF7*, *ISG15*, *ISG20*, *MX1*, *NUP58*, *OAS2*, *OAS3*, *PML*, *RSAD2*, *SOCS*, *STAT2*, *TRIM5*, *TRIM22*, *TRIM35*, *USP18*, *VCAM1*, *XAF1*). A protein-protein interaction network constructed using these overlapping genes is shown in Figure 2C, demonstrating their role within interferon signaling pathways (a minimum network containing 27 of these genes produced from Network Analyst^7^). Examples of inverse correlations between gene expression prior to LAIV challenge and day 2 viral load are shown in Figure 2D (see Table S3 for all correlation coefficients). Children with higher baseline gene expression and lower viral loads were made up mostly, but not exclusively, by those with asymptomatic respiratory viruses.

### Impact of asymptomatic respiratory viruses and upregulated interferon pathways on immunogenicity to LAIV

In keeping with our previous findings of the importance of viral shedding for induction of immunity to LAIV,^5^ significant positive correlations were observed between viral load and increase in HAI titer from baseline to day 21 for H3N2 (day 2 viral load: rho = 0.47, p = 0.002; day 7 viral load: rho = 0.71, p < 0.0001) and influenza B (day 2 viral load: rho = 0.43, p = 0.001; day 7 viral load: rho = 0.56, p < 0.0001), but not for pH1N1 (Figure S1). No associations between viral load and induction of CD4+ T cell responses was observed (Figure S1). No statistically significant difference in either antibody or T cell response was seen to any LAIV strain in children with and without asymptomatic respiratory viruses at baseline (Figure 2E). No significant correlation was also seen between enrichment for interferon genes at baseline and induction of antibody or T cell responses to LAIV (Figure S2).

### Cell-type-specific gene expression signatures associated with LAIV shedding and presence of asymptomatic respiratory viruses

**Figure 3:**
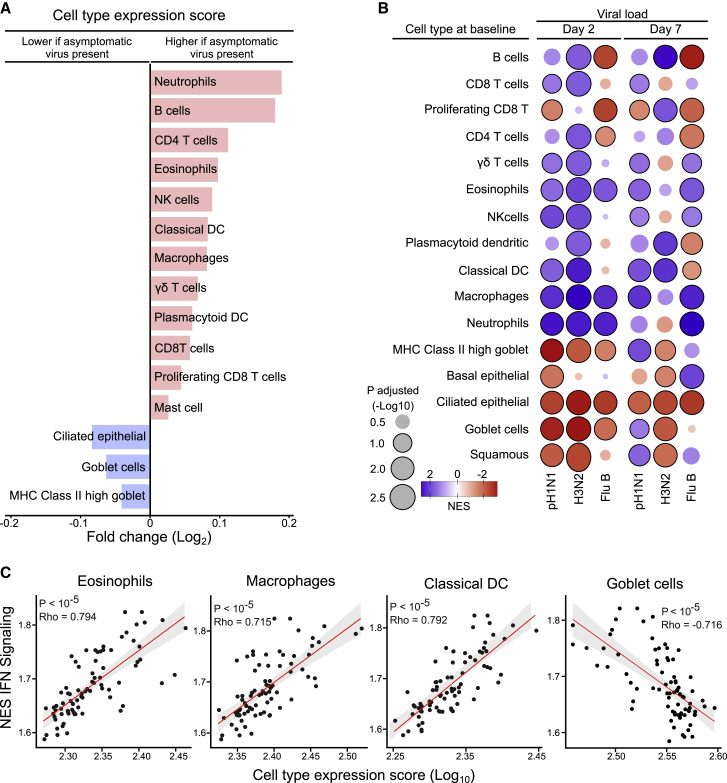
Cell-type-specific gene expression signatures at baseline associated with presence of asymptomatic respiratory viruses and LAIV shedding in children seronegative to each influenza strain prior to vaccination (A) Fold-difference in cell-type-specific gene expression scores for different cell types between children with and without asymptomatic respiratory viruses pre-LAIV challenge. Cell-type-specific expression scores from bulk RNA sequencing (RNA-seq) data were generated using the sum of the normalized expression values from the top 50 genes defining each cell type derived from a previously described nasal wash single-cell RNA-seq data in influenza-infected individuals.^10^ Only cell types with a significant difference between groups (Mann-Whitney U test; Figure S1) are displayed. The fold-difference was calculated using the median values for children positive and negative for asymptomatic respiratory viruses. (B) GSEA using the Spearman correlation coefficients between rlog prior to LAIV challenge and strain-specific viral loads (log_10_ EID_50_/mL) at days 2 and 7 as rank and cell-type-specific gene set as in Cao et al. (2020).^10^ The size of circles is proportional to the adjusted −log_10_ p value from GSEA, while the intensity of the circle color denotes the NES for each cell-type/strain-specific viral load combination (deeper color represents higher NES). Blue circles denote enrichment of genes in nasopharynx prior to LAIV challenge that negatively correlate with viral load at day 2 and day 7, and red circles denote enrichment of genes that positively correlate with viral loads. Significant pathways (adjusted p value < 0.1) are highlighted with a black outline. (C) Correlation between cell-type-specific gene expression scores and NESs for the IFN signaling pathway from single sample GSEA using the Reactome pathway set. Cell-type-specific scores were calculated following exclusion of any genes also present in the IFN signaling pathway gene set to avoid correlation due to these overlapping genes (classical dendritic cells [DCs]: HLA-DP1, HLA-DPB1, HLA-DQA1, HLA-DQB1, HLA-DQB2, HLA-DRB5, IRF8; no overlapping genes for macrophage, eosinophil, or goblet cell gene sets). R, Spearman correlation coefficient.

Comparisons of cell-type-specific expression scores showed that samples from children with asymptomatic respiratory viruses were significantly enriched for signatures from a variety of immune cells (e.g., neutrophils, eosinophils, macrophages), with concomitant reduction in those gene signatures from ciliated epithelial cells, goblet cells, and major histocompatibility complex (MHC) class II high goblet cells (Figure 3A; Figure S3). The reduction in the proportion of multiciliated epithelial cell signatures in children with respiratory viruses (p = 5e^−3^) was confirmed by a deconvolution analysis based on single-cell data from healthy human airways (Figure S4).^8^^,^^9^

To determine the relationship between enrichment for cell-type-specific signatures prior to LAIV challenge and subsequent viral load at days 2 and 7, GSEA was repeated using gene viral load correlation coefficients as rank and the cell-specific gene list as the reference set.^10^ Significant enrichment for neutrophil, eosinophil, and macrophage signatures pre-challenge was associated with lower viral load at day 2 for all three strains (Figure 3B; Table S4). Significant enrichment for ciliated epithelial cell signatures pre-challenge was associated with higher viral loads at both day 2 and day 7 for all three strains. Finally, to explore how enrichment for specific cells may be linked to interferon signaling pathway upregulation, correlations between cell-specific scores and interferon pathway NES from single-sample GSEA were conducted. Significant positive correlations were observed between interferon pathway enrichment and signatures for several cell types (e.g., eosinophils, macrophages, natural killer [NK] cells), with significant inverse correlations with ciliated epithelial cells, goblet cells, and MHC class II high goblet cells (Figure 3C; Figure S5).

## Discussion

Using live attenuated influenza strains as a surrogate controlled influenza challenge model in young children, we demonstrate that pre-existing upregulated interferon pathways in the URT mucosa may limit the replication of influenza viruses in seronegative children. Furthermore, we show that these changes may in part be due to asymptomatic infection with other respiratory viruses, highlighting the complex interplay between the large burden of viruses that children are exposed to. Our findings have implications not only for understanding the reasons for the heterogenous outcomes from pediatric influenza infections^11^ but also more broadly for investigating the divergent outcomes from other respiratory viral infections such as severe acute respiratory syndrome coronavirus 2 (SARS-CoV-2) in children.

The ability of interferon to inhibit influenza virus replication in humans through the induction of ISGs is well described.^12^ Our findings are consistent with recent *in vitro* data from a co-culture model showing inhibition of pdm09H1N1 influenza virus replication by preceding human rhinovirus infection via ISG induction.^13^ This was suggested as a potential explanation for the epidemiological observations of asynchronous circulation of rhinovirus and influenza A viruses.^14^ Interference with influenza infections by other viral co-infections (CoVs, respiratory syncytial virus [RSV]) has also been shown in murine and ferret models.^15^^,^^16^ Our data clearly demonstrate that asymptomatic respiratory viral infections are common in children and can cause perturbation of the nasal mucosal transcriptome. While a previous study found blood transcriptional responses in children asymptomatic rhinovirus infection were no different to healthy controls,^17^ another study with limited asymptomatic cases found alterations in nasal samples.^18^ While beyond the scope of this study, it is possible that colonizing bacteria and fungi also impact influenza virus replication, potentially via trained immunity, and should be explored in future cohorts.

Although inferred from transcriptional signatures alone, our data also suggest that the composition of cell types in the nasopharyngeal mucosa at the time of LAIV infection can impact viral replication in the first 2 days. Children with baseline gene signatures enriched in macrophages, neutrophils, and eosinophils were significantly more likely to have lower viral loads at day 2 for all three influenza strains. Macrophages, in particular, are known to play a key role in interferon induction and control of influenza infections.^19^ We also found that signatures from macrophages, neutrophils, and eosinophils, as well as a range of other immune cells, were upregulated in children with asymptomatic viral infections. Significant correlations were seen between several of these cell-type signatures and interferon signaling pathways.

Interestingly, we observed upregulation of pathways associated with ciliary function (Intraflagellar transport, Anchoring of the basal body to the plasma membrane) at baseline were associated with higher LAIV viral loads. In addition, pre-challenge enrichment for a gene set representative of ciliated epithelial cells was significantly associated with higher viral loads at later time points. Asymptomatic viral infection also resulted in reduction in cell-type expression scores for several epithelial cells, but notably for ciliated epithelial cells. This could simply represent a scenario where loss of epithelial cells due to preceding viral infection results in a reduction in preferential target cells for influenza infection. Children with greater proportions of ciliated epithelial cells at the time of infection may end up having higher influenza viral loads. It is also possible that the infiltration of other cells during viral infection leads to a lower proportion of epithelial cells within the total cells sampled or that variability in nasopharyngeal swabbing may have contributed to our observed result. However, previous data from human airway epithelial cells have shown that human rhinovirus infection downregulates several genes associated with cilium movement and morphogenesis,^20^ with several of these found in the Intraflagellar transport pathway leading edge genes in our analysis (e.g., *DYNC2H1*, *DYNC2LI1*, *IFT20*, *IFT88*, *IFT122*, *IFT172*, *KIF3A*, *TTC26*, *WDR35*). These findings warrant further *in vitro* exploration to understand the role of ciliary function genes in facilitating influenza replication.

Our findings would benefit from being replicated in other studies. Little is known about the variability in mucosal gene expression in children classified as healthy from diverse geographical, environmental, and socioeconomic settings. The burden of asymptomatic respiratory viruses in children will vary seasonally in the same location as well as between countries. Nevertheless, it is biologically plausible that a similar relationship will be seen between upregulated interferon pathways and impaired replication of LAIV strains regardless of where children reside. Reassuringly, we did not see any negative impact of asymptomatic viral infection or upregulated interferon gene expression pre-vaccination on antibody and T cell responses to LAIV. While we have shown that shedding of LAIV strains at day 7 is significantly associated with higher HAI and CD4+ T cell responses to LAIV,^5^ our current data suggest that the reduced viral replication associated with pre-upregulated interferon in some children is not sufficient to significantly impair this immunogenicity.

We present proof of concept that LAIV could be used as a surrogate influenza challenge model in young children to understand host-virus and virus-virus interactions in the upper respiratory tract. Mucosal transcriptomic studies in pediatric influenza infection to date are limited to sampling after acute presentation, where pre-infection factors cannot be determined. Our methodology can also be applied to future pediatric studies to obtain detailed nasopharyngeal host and pathogen data from minimally invasive sampling. Integrating such methods into household viral transmission studies with systematic sampling over time should be used to expand our experimental findings with LAIV to other respiratory viruses. This could be particularly relevant in exploring why children appear to be less susceptible to symptomatic SARS-CoV-2 infection and determining whether mucosal factors such as upregulated ISGs prior to exposure impact acquisition, severity of infection and onward transmission.

### Limitations of the study

The major limitation of our study is that only transcriptional signatures were assessed rather than protein and cytokine measurement, or cellular characterization via flow cytometry. Challenges to sampling in young children limit the ability to undertake extensive investigation, but it would be important to validate our findings in future studies. Although we documented asymptomatic viral infections at baseline by PCR in more than 40% of children, it is difficult to know when these children were infected as viral nucleic acid can be detected for prolonged periods after acute infection. This may in part explain the heterogeneity in interferon gene upregulation seen in children with asymptomatic respiratory viral infections. It is also not known whether the attenuating mutations in LAIV strains impact their susceptibility to antiviral interferons, which may affect to what degree our findings are generalizable to wild-type influenza strains. Despite producing significantly less infectious virus, LAIV strains have been shown to induce greater antiviral innate responses *in vitro* than antigen-matched seasonal influenza viruses, which may contribute to the attenuated phenotype observed.^3^

## STAR★Methods

### Key resources table

**Table:** 

REAGENT or RESOURCE	SOURCE	IDENTIFIER
**Antibodies**		

CD4 PerCPCy5.5-conjugated antibody	Biolegend	317428; RRID:AB_1186122
CD8 FITC-conjugated antibody	Biolegend	301050; RRID:AB_2562055
IL-2 PE-conjugated antibody	Biolegend	500307; RRID:AB_315094
IFN- γ APC-conjugated antibody	Biolegend	506510; RRID:AB_315443
Purified mouse anti-human CD49d	BD Biosciences	340976; RRID:AB_400198
Purified mouse anti-human CD28	BD Biosciences	340975; RRID:AB_400197
BD Golgiplug (Protein Transport Inhibitor – Brefeldin A)	BD Biosciences	555029; RRID:AB_2869014
Anti-Rat Ig, negative control compensation particles set	BD Biosciences	552844; RRID:AB_10055784)
Anti-Mouse Ig, negative control compensation particles set	BD Biosciences	552843; RRID:AB_10051478
ARCTM Amine reactive compensation bead kit (for Live/Dead staining)	Molecular Probes	A10346
Zombie Violet Fixable Viability Kit	Biolegend	423114
Staphylococcal Enterotoxin B	Sigma Aldrich	S4811

**Bacterial and virus strains**		

Equine Arteritis Virus	RIVM, the Netherlands (in-house)	N/A
A/Michigan/45/2015(H1N1) pdm09-like virus	Worldwide Influenza Centre at the Crick Institute (London, UK)	N/A
A/HongKong/4801/2014(H3N2)-like virus	Worldwide Influenza Centre at the Crick Institute (London, UK)	N/A
A/California/7/2009(H1N1)pdm09-like virus	Worldwide Influenza Centre at the Crick Institute (London, UK)	N/A
B/Brisbane/60/2008-like virus	Worldwide Influenza Centre at the Crick Institute (London, UK)	N/A
B/Texas/02/2013)-like virus	Worldwide Influenza Centre at the Crick Institute (London, UK)	N/A

**Biological samples**		

Specific ferret post-infection antisera as controls	In-House	N/A

**Chemicals, peptides, and recombinant proteins**		

Phosphate Buffered Saline (PBS) tablets	Sigma	P4417 100TAB
Sterile distilled water	N/A	N/A
70% Ethanol	Variable	Variable
Pen/strep	Variable	Variable
RPMI	Variable	Variable
Foetal Bovine Serum (FBS)	GIBCO-Life Technologies	10500-064
Sodium Azide	Sigma Aldrich	S2002-25G
20mM EDTA	Sigma Aldrich	E7889-100ml
L- Glutamine	Variable	Variable
Influenza overlapping peptide pools (Matrix and Nucleoprotein – MNP, H1 haemagglutinin – HA1, H3 haemagglutinin – HA3, Influenza B Haemagglutinin- HA, Influenza B Matrix and Nucleoprotein - MNP) reconstituted in DMSO and stored at −70°C (so final concentration in assay is 2 μg/ml)	Sigma-Aldrich	Custom
Propan-2-ol (isopropanol) – molecular grade 96 −100%	Various	Various
Chloroform	Various	Various
Ethanol – molecular grade 96 −100%	Various	Various
RNAprotect Cell Reagent	QIAGEN	76526
RNase Zap reagent	Various	Various
PBS (1X), sterile liquid; pH 7.2 ± 0.05 (-CaCl2, -MgCl2)	GIBCO Invitrogen	20012-019
Physiological Saline; Sodium chloride, Tablet 1/100mL	Sigma Aldrich	S6150-50TAB
HPLC graded distilled water	VWR	83645.290
Diethyl ether ACS reagent, anhydrous, ≥ 99.0%, contains BHT as inhibitor	Sigma Aldrich	346136-250ML
TWEEN® 80 10% Low-peroxide	Sigma Aldrich	P8192-10ML
Receptor Destroying Enzyme (RDE)	Accurate Chemical	YCC340122
Bovine serum albumin (BSA)	Sigma	A8327
Oseltamivir Carboxylate	Roche	GS4071 / Ro64-0802

**Critical commercial assays**		

QubitTM RNA HS Assay kit	QIAGEN	Q32852
SPLIT RNA Extraction Kit	Lexogen	008
QuantSeq 3′ mRNA-Seq Library Prep Kit	Lexogen	015.24
QIAamp Cador Pathogen mini kit (Indispin pathogen kit)	Indical Bioscience Gmbh, Germany	54106
Copan pediatric flocked eSwab with Amie’s medium	Copan	484CE
Copan Universal Transport Medium collection kit with nasopharyngeal flocked swab	Copan	360C
Copan empty tube for E-Swab		PFPM913S

**Deposited data**		

Raw and processed bulk RNA-seq data	This paper	GEO: GSE159884
Analyses and resources: scripts and codes	This paper, Github data	https://github.com/csbl-br/Mucosal_transcriptome_LAIV_shedding

**Experimental models: Cell lines**		

0.5% Turkey erythrocytes	In-house (Public Health England)	NA
0.5% Guinea Pig erythrocytes	In-house (Public Health England)	NA

**Multiplex viral assay – Reagents and chemicals**		

Invitrogen Superscript III platinum 1 step RT PCR kit	ThermoFisher Scientific	12574018
Platinum Taq DNA Polymerase, DNA-free	ThermoFisher Scientific	15966005

**Multiplex viral assay – Oligonucleotides**		

influenza virus A forward primer	Eurogentec	AAGACAAGACCAATYCTGTCACCTCT
influenza virus A reverse primer	Eurogentec	TCTACGYTGCAGTCCYCGCT
influenza virus A probe	Eurogentec	FAM-TYACGCTCACCGTGCCCAGTG-BHQ1
influenza virus B forward primer	Eurogentec	ATGATCTTACAGTGGAGGATGAAGAA
influenza virus B reverse primer	Eurogentec	CGAATTGGCTTTGRATGTCCTT
influenza virus B probe	Eurogentec	CY5-ATGGCCATCGGATCCTCAAYTC ACTCT-BHQ1
parainfluenza virus 1 forward primer	Eurogentec	GTGATTTAAACCCGGTAATTTCTCA
parainfluenza virus 1 reverse primer	Eurogentec	CCTTGTTCCTGCAGCTATTACAGA
parainfluenza virus 1 probe	Eurogentec	FAM-ACCTATGACATCAACGAC-BHQ2
parainfluenza virus 2 forward primer	Eurogentec	ATGAAAACCATTTACCTAAGTGATGGA
parainfluenza virus 2 reverse primer	Eurogentec	CCTCCYGGTATRGCAGTGACTGAAC
parainfluenza virus 2 probe	Eurogentec	VIC-TCAATCGCAAAAGC-BHQ2
parainfluenza virus 3 forward primer	Eurogentec	CCAGGGATATAYTAYAAAGGCAAAA
parainfluenza virus 3 reverse primer	Eurogentec	CCGGGRCACCCAGTTGTG
parainfluenza virus 3 probe	Eurogentec	FAM-TGGRTGTTCAAGACCTCCATA YCCGAGAAA-BHQ1
parainfluenza virus 4 forward primer	Eurogentec	CAGAYAACATCAATCGCCTTACAAA
parainfluenza virus 4 reverse primer	Eurogentec	TGTACCTATGACTGCCCCAAARA
parainfluenza virus 4 probe	Eurogentec	CY5-CCMATCACAAGCTCAGAAAT YCAAAGTCGT-BHQ1
Human coronavirus 229E forward primer	Eurogentec	CAGTCAAATGGGCTGATGCA
Human coronavirus 229E reverse primer	Eurogentec	AAAGGGCTATAAAGAGAATAAGGTATTCT
Human coronavirus 229E probe	Eurogentec	FAM-CCCTGACGACCACGTTGTGGTTCA-BHQ1
Human coronavirus OC43 forward primer	Eurogentec	CCTTCCTGAGCCTTCAATATAGTAACC
Human coronavirus OC43 reverse primer	Eurogentec	CGATGAGGCTATTCCGACTAGGT
Human coronavirus OC43 probe	Eurogentec	FAM-TCCGCCTGGCACGGTACTCCCT-BHQ1
Human coronavirus NL63 forward primer	Eurogentec	ACGTACTTCTATTATGAAGCATGATATTAA
Human coronavirus NL63 reverse primer	Eurogentec	AGCAGATCTAATGTTATACTTAAAACTACG
Human coronavirus NL63 probe	Eurogentec	FAM-ATTGCCAAGGCTCCTAAACGTAC AGGTGTT-BHQ1
rhinovirus forward primer	Eurogentec	TGGACAGGGTGTGAAGAGC
rhinovirus reverse primer	Eurogentec	CAAAGTAGTCGGTCCCATCC
rhinovirus probe	Eurogentec	HEX-TCCTCCGGCCCCTGAATG-BHQ1
Respiratory syncytial virus A forward primer	Eurogentec	AGATCAACTTCTGTCATCCAGCAA
Respiratory syncytial virus A reverse primer	Eurogentec	TTCTGCACATCATAATTAGGAG
Respiratory syncytial virus A probe	Eurogentec	FAM-CACCATCCAACGGAGCACAGGAGAT-BHQ1
Respiratory syncytial virus B forward primer	Eurogentec	AAGATGCAAATCATAAATTCACAGGA
Respiratory syncytial virus B reverse primer	Eurogentec	TGATATCCAGCATCTTTAAGTA
Respiratory syncytial virus B probe	Eurogentec	CY5-TTTCCCTTCCTAACCTGGACATA-BHQ1
Swine Flu forward primer	Eurogentec	TGTGCCACTTGTGAACAGATTG
Swine Flu reverse primer	Eurogentec	CTGATTAGTGGATTGGTGGTAGTAGC
Swine Flu probe	Eurogentec	HEX-TGATTCACAGCATCGGTCTC ACAGACAG-BHQ1
EVA (RNA ic) forward primer	Eurogentec	CTGTCGCTTGTGCTCAATTTAC
EVA (RNA ic) reverse primer	Eurogentec	AGCGTCCGAAGCATCTC
EVA (RNA ic) probe	Eurogentec	ROX-TGCAGCTTATGTTCCTTGCA CTGTGTTC-BHQ1
Adenovirus forward primer	Eurogentec	GCCACGGTGGGGTTTCTAAACTT
Adenovirus reverse primer	Eurogentec	GCCCCAGTGGTCTTACATGCACATC
Adenovirus probe	Eurogentec	FAM-TGCACCAGACCCGGGCTCAGGT ACTCCGA-BHQ1
Human Metapneumovirus A forward primer	Eurogentec	GCYGTYAGCTTCAGTCAATTCAA
Human Metapneumovirus A reverse primer	Eurogentec	TCCAGCATTGTCTGAAAATTGC
Human Metapneumovirus A probe	Eurogentec	VIC-CAACATTTAGAAACCTTCT-BHQ1
Human Metapneumovirus B forward primer	Eurogentec	GCYGTYAGCTTCAGTCAATTCAA (Common with A)
Human Metapneumovirus B reverse primer	Eurogentec	GTTATCCCTGCATTGTCTGAAAACT
Human Metapneumovirus B probe	Eurogentec	VIC-CGCACAACATTTAGGAATCTTCT-BHQ1

**Software and algorithms**		

Picard	Picard	http://broadinstitute.github.io/picard/
UMI-tools	UMI-tools	https://umi-tools.readthedocs.io/en/latest/index.html
DART	Lin and Hsu, 2018	https://github.com/hsinnan75/Dart
Flowjo v10.7.1	BD Biosciences	N/A
Cytoscape v3.8.0	Cytoscape	https://cytoscape.org/
NetworkAnalyst v3.0	Zhou et al., 2019^7^	https://www.networkanalyst.ca/
fgsea R package v1.10.1	Korotkevich et al., 2019^21^	http://bioconductor.org/packages/release/bioc/html/fgsea.html
FastQC	Bioinformatics	https://www.bioinformatics.babraham.ac.uk/projects/fastqc/
Rsubread R package	Bioconductor	N/A
DESeq2 R package v1.24.0	Love et al., 2014^22^	https://bioconductor.org/packages/release/bioc/html/DESeq2.html
ComplexHeatmap R package v2.0.0	Bioconductor	https://bioconductor.org/packages/release/bioc/html/ComplexHeatmap.html
Ggplot2 R package 3.3.5	CRAN	https://cran.r-project.org/web/packages/ggplot2/index.html
EnhancedVolcano R package v1.2.0	Bioconductor	https://bioconductor.org/packages/release/bioc/html/EnhancedVolcano.html
BiocParallel R package	Bioconductor	https://bioconductor.org/packages/release/bioc/html/BiocParallel.html
MuSIC R package v0.1.1	Wang et al., 2019^^9^^	https://xuranw.github.io/MuSiC/index.html

**Other**		

BD LSR II Fortessa	BD Biosciences UK	N/A
QubitTM Flurometer	QIAGEN	Q33238
Illumina NextSeq 500	Illumina	NA
Single-cell Data (Single-cell atlas of the airway epithelium)	Deprez et al., 2020^8^	https://www.genomique.eu/cellbrowser/HCA/
Pathway gene set data (Reactome)	Reactome	https://reactome.org/download-data

### Resource availability

#### Lead contact

Further information and requests for resources and reagents should be directed to and will be fulfilled by the lead contact, Thushan de Silva (t.desilva@sheffield.ac.uk).

#### Materials availability

This study did not generate any new unique reagents available for distribution.

### Experimental model and subject details

#### Study Design, sampling and LAIV inoculation

Influenza-vaccine naive children aged 24-59 months were recruited and administered one dose of trivalent LAIV as previously described (median age 35 months, 44% female).^5^ Inclusion and exclusion criteria for the study were as follows:

#### Inclusion Criteria

• Healthy male or female child at least 24 months of age and less than 60 months of age at the time of study entry.
• Resident in the study area and with no plans to travel outside the study area during the period of subject participation.
• Informed consent for the study participation obtained from a parent (or guardian only if neither parent is alive or if guardianship has been legally transferred).
• Willingness and capacity to comply with the study protocol as judged by a member of the clinical trial team.

#### Exclusion Criteria

• Serious, active, medical condition, including but not limited to:
• chronic disease of any body system
• severe protein-energy malnutrition (weight-for-height Z-score of less than −3)
• known genetic disorders, such as Down syndrome or other cytogenetic disorder
• Active wheezing
• History of documented hypersensitivity to eggs or other components of the vaccine (including gelatin, sorbitol, lactalbumin and chicken protein), or with life-threatening reactions to previous influenza vaccinations.
• History of documented hypersensitivity to macrolide antibiotics
• History of Guillain-Barré syndrome.
• Receipt of aspirin therapy or aspirin-containing therapy within the two weeks before planned study vaccination.
• Any suspected or confirmed congenital or acquired state of immune deficiency including but not limited to primary immunodeficiencies including thymus disorders, HIV/AIDS, hematological or lymphoid malignancies.
• Any current immunosuppressive/immunomodulatory treatment or receipt of any such treatment within the six months preceding trial enrolment (for corticosteroids this is defined as a dose of prednisolone (or equivalent) of greater than 2mg/kg/day for one week or 1mg/kg/day for one month. The use of topical corticosteroids is not an exclusion criterion.
• The use of inhaled corticosteroids within the last one month.
• Receipt of an influenza vaccine within the past 12 months.
• Has any condition determined by investigator as likely to interfere with evaluation of the vaccine or be a significant potential health risk to the child or make it unlikely that the child would complete the study?
• Any significant signs or symptoms of an acute illness or infection including:
• an axillary temperature of 38.0°C or above or documented fever of 38°C or above in the preceding 14 days.
• Any acute respiratory infection within 14 days of enrolment visit.

Only children given the 2017-18 northern hemisphere LAIV formulation were included in the current analysis (administered Feb – April 2018), as this vaccine included three influenza strains with good replicative ability following replacement of the A/California/07/2009-like pH1N1 strain from previous years.^5^ Influenza titers per dose (50% Egg Infectious Doses (EID_50_)/ml) were 1x10^7.7^ for pH1N1 (A/17/New York/15/5364), 1x10^7.6^ for H3N2 (A/17/Hong Kong/2014/8296) and 1x10^7.3^ for the influenza B Victoria-lineage strain (B/Texas/02/2013).

Nasopharyngeal swabs (NPS) were taken pre-LAIV challenge, and at day 2 (D2) and day 7 (D7), using flocked swabs (Copan FloQSwabs™) collected directly into RNAprotect Cell Reagent (QIAGEN). Samples were transported at 2-8°C and stored at −70°C within 4 hours of collection. Whole blood was collected pre-LAIV, serum separated and stored at −70°C prior to further processing.

#### Ethical approval and consent

The study was approved by the Gambia Government/MRC Joint Ethics Committee (SCC1502) and the Medicines Control Agency of The Gambia, and conducted according to ICH-GCP standards. Parents provided written or thumbprinted informed consent for their children to participate.

### Method details

#### Antibody and T cell responses to LAIV

Haemagglutinin inhibition (HAI) assays were performed according to standard methods, using vaccine haemagglutinin (HA) and neuraminidase-matched viruses.^23^ Children with baseline HAI titer < 1:10 were classified as seronegative to a given influenza strain. HAI geometric mean titers were measured before and after (day 21) LAIV, allowing calculation of a geometric mean fold rise (GMFR) following vaccination. Haemagglutinin-specific CD4+ T cell responses before and after vaccination were measured using intra-cellular cytokine staining following stimulation with overlapping peptides representing haemagglutinin proteins of vaccine-matched pH1N1, H3N2 and influenza B strains as previously described.^5^ A fold change in the percentage of CD4+ T cells expressing interferon-gamma at day 21 compared to day 0 was estimated to evaluate vaccine responses.

#### Detection of Respiratory Viruses & quantitative RT-PCR for LAIV strains

Total nucleic acid was extracted from 180μL of RNAprotect using the QIAamp Cador Pathogen mini kit (Indical Bioscience Gmbh, Germany) as per manufacturer’s instructions. All samples were spiked with Equine Arteritis Virus as an extraction control. A previously described multiplex reverse-transcriptase polymerase-chain reaction (RT-PCR) was performed to detect influenza A, influenza B, Respiratory Syncytial Virus (RSV) A, RSV B, human parainfluenza viruses (HPIV) 1 to 4, human metapneumovirus, adenovirus, seasonal coronaviruses (CoV; 229E, OC43, NL63) and human rhinovirus.^6^

Total nucleic acid was extracted from 270μL of RNAprotect as described above. LAIV strain viral loads were quantified as previously described, with monoplex HA-specific RT-PCRs using a standard curve with known log10 50% EID/ml.^5^ RT-PCR negative samples were assigned a log10 50% EID/ml based on the lower limit of detection of each assay (−0.219 for pH1N1, −0.319 for H3N2 and −0.619 for influenza B) for the purpose of analyses described below.

#### RNA-sequencing of nasopharyngeal samples and data processing

RNA was extracted from 400μL of RNAprotect using the SPLIT RNA Extraction Kit (Lexogen) as per manufacturer’s instructions. Samples with at least 100ng of RNA were taken forward for QuantSeq 3′ messenger RNA sequencing (Lexogen).^24^ QuantSeq libraries were prepared using the QuantSeq 3′ mRNA-Seq Library Prep Kit for Illumina according to manufacturer’s instructions, with the addition of unique molecular identifiers for exclusion of PCR duplicates. Libraries were sequenced using the Illumina NextSeq 500 to produce 75bp single-end reads for each sample.

Raw data quality control was performed using FastQC (Bioinformatics). Mapping to the human reference genome (GRCh38/hg38) was done using DART (Lin and Hsu, 2018). PCR duplicates were removed using umi_tools dedup in Python 3.7.2 and optical duplicates removed using the Picard MarkDuplicates tool (http://broadinstitute.github.io/picard/). Reads were counted at gene level using the Rsubread R package.^25^ For genes with multiple isoforms, only the transcript which displayed the highest mean expression between all samples was selected. Raw and processed data are available in the GEO database (GSE159884). Genes with counts < 30 were excluded (leaving 32932 genes) and the data normalized using rlog and varianceStabilizingTransformation (VST) functions from DESeq2 Bioconductor package.^22^

Cell type-specific expression scores from bulk RNA-seq data were generated using the sum of the VST-normalized expression values from the top 50 genes defining each cell type derived from a previously described nasal wash single-cell RNaseq data in influenza-infected individuals.^10^ Deconvolution of bulk RNA-seq was also performed using the MuSIC package,^9^ with single-cell atlas data from human healthy airway tissue as the reference set.^8^ The scores for each cell type were then compared between children with and without asymptomatic respiratory viruses, as a surrogate for determining how these infections may affect the presence of different cell types in the nasopharyngeal mucosa.

### Quantification and statistical analysis

#### Bioinformatic analysis

Analyses were conducted in R software version 3.6. Gene set enrichment analyses (GSEA) were conducted using the fgsea Bioconductor package.^21^ A list of Spearman rank correlation coefficients between LAIV viral loads and rlog normalized expression for each gene was used as a rank. Enrichment analyses were performed using the Reactome pathway set and, in a separate analysis, the cell-subset marker set (50 defining genes/subset).^10^ Single sample GSEA using baseline gene expression scores as rank for each individual was also conducted for Reactome pathways. Normalized enrichment scores (NES) and adjusted *P*-values for each pathway/cell type were extracted, along with leading edge genes in each set. Pathways with an adjusted *P*-value of < 0.1 were considered significant, which is more conservative than the adjusted *P*-value threshold of 0.25 used previously in GSEA.^26^

DESeq2 Bioconductor was used to detect differentially expressed genes (DEG) between children with and without asymptomatic viral infections prior to LAIV challenge.^22^ A false discovery rate correction using the Benjamini-Hochberg method was used as standard for DEG analyses to generate adjusted *P*-values for each gene. A log2 fold-change of ± 0.5 and a stringent adjusted *P*-value of 0.001 were used to select DEG for further consideration.

Networks were constructed with NetworkAnalyst 3.0 using InnateDB protein-protein interaction data and the minimum network option.^7^ Networks were edited and visualized using Cytoscape (https://cytoscape.org/).

#### Statistical analysis

Comparisons of continuous data between groups were performed using the Mann Whitney U test. All correlations were estimated using Spearman’s rank correlation coefficients. Analyses were conducted in R software version 3.6.

## Data Availability

Code and data used for performing Spearman correlation between viral load data and gene or pathway expression, related to all figures, are public available at Github repository (https://github.com/csbl-br/Mucosal_transcriptome_LAIV_shedding). The analysis folder contains numbered R and Bash scripts used for data manipulation in one subfolder: data/raw to be used as script inputs. Processed files and plots should be obtained by executing script sequentially (order is indicated by file number) using the provided data as inputs. Code and data used for plotting corrplots, heatmap, volcano and scatterplots are provided within numbered R scripts. The plots were manually edited and assembled using Inkscape to improve presentation (https://inkscape.org/) to form the manuscript figures. Any additional information required to re-analyze the data reported in this paper is available from the lead contact upon request.

## Supplemental information

Document S1. Figures S1–S5

Table S1. Significant Reactome pathways from gene set enrichment analysis associated with viral load at day 2 and day 7, related to Figure 2

Table S2. Differentially expressed genes between children with and without asymptomatic respiratory viral infections, related to Figure 2

Table S3. Correlation between mucosal gene expression prior to LAIV receipt and LAIV strain viral loads at days 2 and 7, related to Figure 2

Table S4. Significant cell-type gene sets from gene set enrichment analysis associated with viral load at day 2 and day 7, related to Figure 3

Document S2. Article plus supplemental information
