# Prior upregulation of interferon pathways in the nasopharynx impacts viral shedding following live attenuated influenza vaccine challenge in children

**DOI:** 10.1016/j.xcrm.2022.100516

**Published:** 2022-02-15

**Authors:** André G. Costa-Martins, Karim Mane, Benjamin B. Lindsey, Rodrigo L.T. Ogava, I′caro Castro, Ya Jankey Jagne, Hadijatou J. Sallah, Edwin P. Armitage, Sheikh Jarju, Bankole Ahadzie, Rebecca Ellis-Watson, John S. Tregoning, Colin D. Bingle, Debby Bogaert, Ed Clarke, Jose Ordovas-Montanes, David Jeffries, Beate Kampmann, Helder I. Nakaya, Thushan I. de Silva

## Main text

(Cell Reports Medicine *2*, 100465; December 21, 2021)

In the originally published version of the manuscript by Costa-Martins et al., Figure 3B contained two errors. First, the numerical values on the color-gradient axis for NES values were accidentally flipped, mistakenly indicating that blue corresponded to an NES value of 2, while red corresponded to an NES value of −2, when, in fact, the opposite should have been indicated (with blue corresponding to −2 and red corresponding to 2). Second, some of the circles that represented viral load values were misshapen. The correct values now appear above the color-gradient bar in Figure 3B, and the shapes of the circles have been corrected in the article online. The authors and Cell Press apologize for the confusion.

**Figure:**
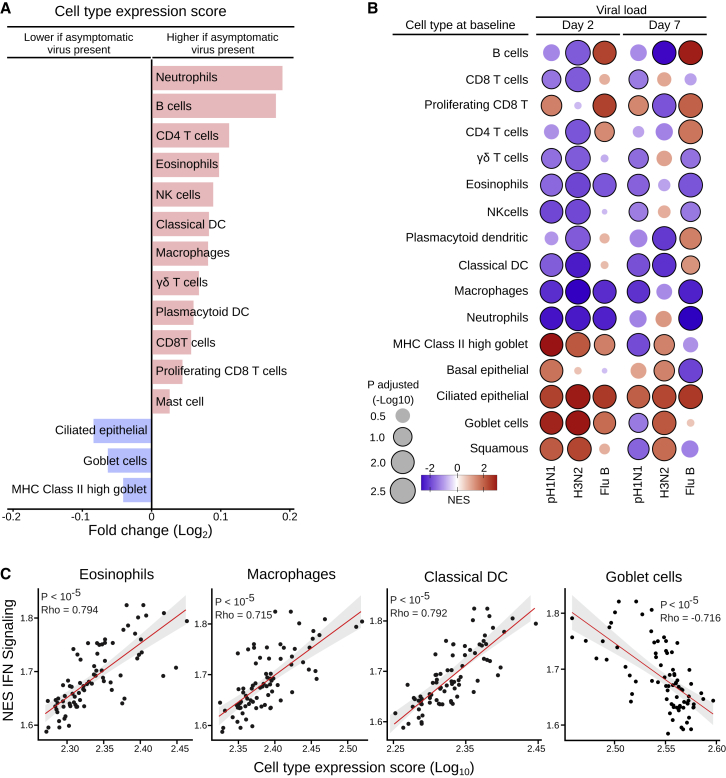
Figure 3. Cell-type-specific gene expression signatures at baseline associated with presence of asymptomatic respiratory viruses and LAIV shedding in children seronegative to each influenza strain prior to vaccination (corrected)

**Figure:**
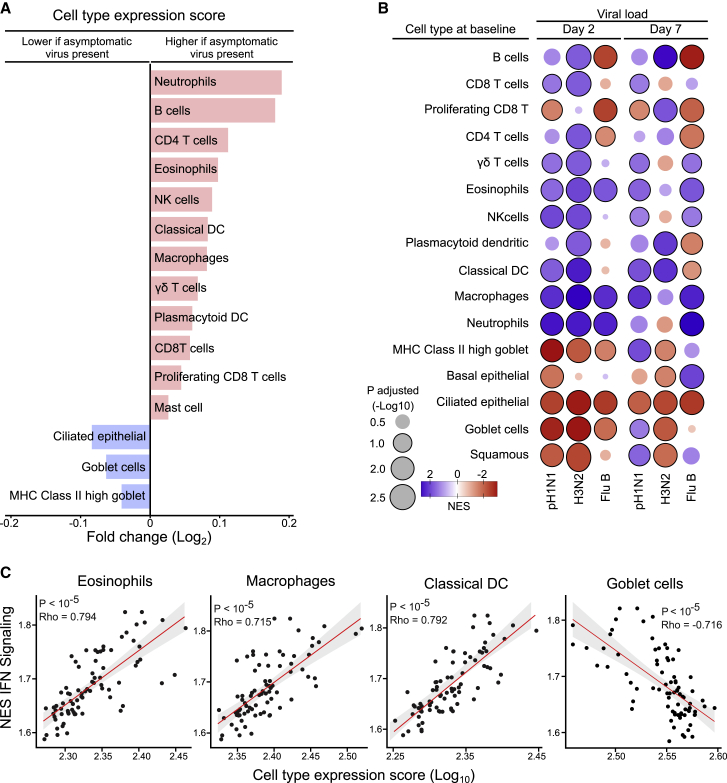
Figure 3. Cell-type-specific gene expression signatures at baseline associated with presence of asymptomatic respiratory viruses and LAIV shedding in children seronegative to each influenza strain prior to vaccination (original)

