# Supplementary material for: Prior upregulation of interferon pathways in the nasopharynx impacts viral shedding following live attenuated influenza vaccine challenge in children
Source: Cell Rep Med. 2021 Dec 9;2(12):100465. doi: 10.1016/j.xcrm.2021.100465 (PMC8714852; doi:10.1016/j.xcrm.2021.100465)
Supplement: Document S1. Figures S1–S5 [file mmc1.pdf]

Cell Reports Medicine, Volume *2*

Supplemental information

Prior upregulation of interferon pathways in the nasopharynx impacts viral shedding following live attenuated influenza vaccine challenge in children

André G. Costa-Martins, Karim Mane, Benjamin B. Lindsey, Rodrigo L.T. Ogava, Ícaro Castro, Ya Jankey Jagne, Hadijatou J. Sallah, Edwin P. Armitage, Sheikh Jarju, Bankole Ahadzie, Rebecca Ellis-Watson, John S. Tregoning, Colin D. Bingle, Debby Bogaert, Ed Clarke, Jose Ordovas-Montanes, David Jeffries, Beate Kampmann, Helder I. Nakaya, and Thushan I. de Silva

SUPPLEMENTARY INFORMATION

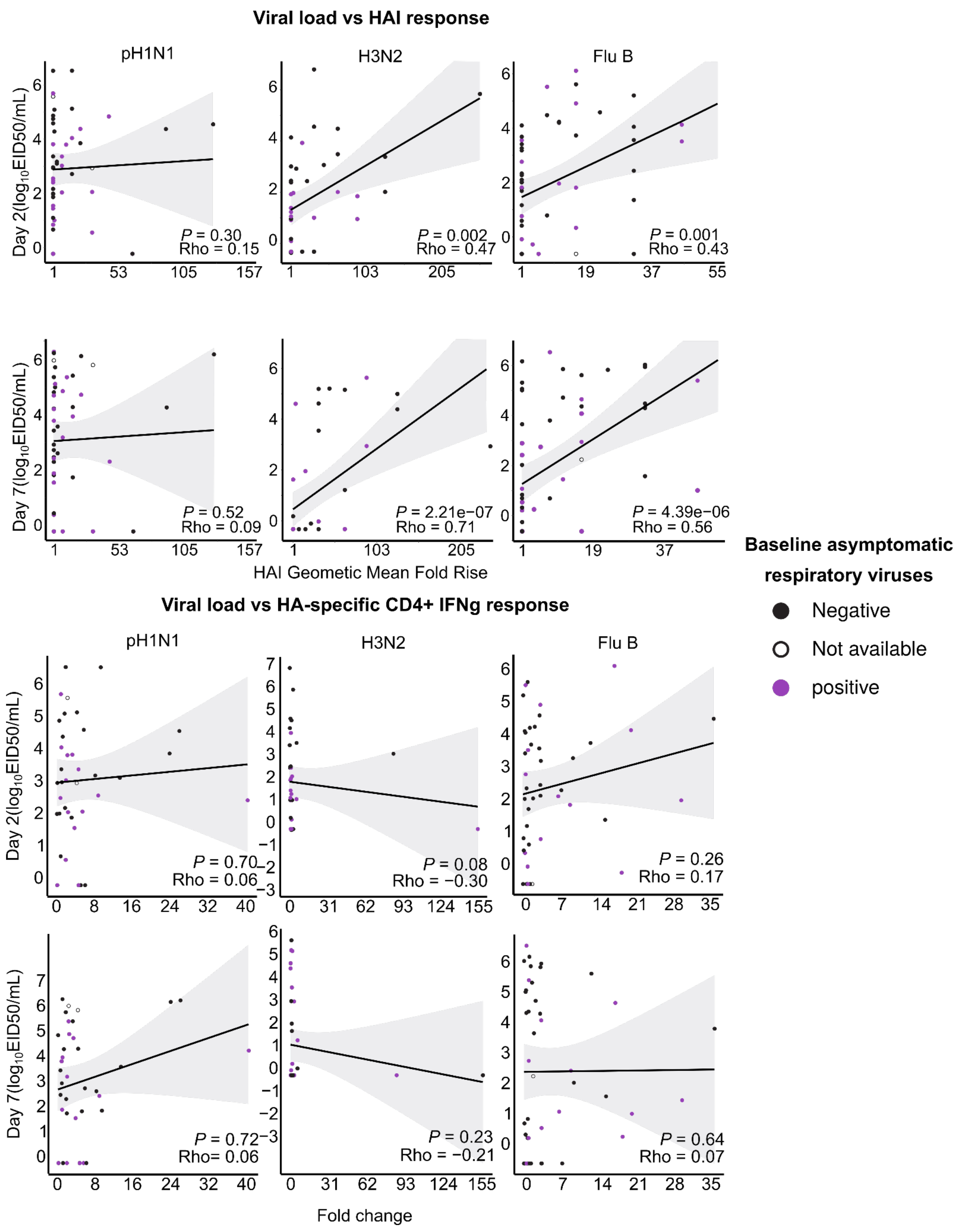

Supplementary figure 1. Relationships between LAIV strain viral load at day 2 or 7 following LAIV and immune response at day 21 after vaccination; related to Figure 2. Antibody response is expressed as the fold rise in the haemagglutinin inhibition (HAI) geometric mean titre after vaccination (geometric mean fold rise) and CD4+ T-cell response as the fold change after vaccination in the percentage of CD4+ T-cells expressing interferon gamma (IFNg) following stimulation with strain-specific haemagglutinin (HA) peptides. Children with asymptomatic viruses detected at baseline are colored in purple. Statistical comparisons performed with Spearman's rank correlation coefficients.

Interferon Signaling		
**Variable**	**Rho**	**Adjusted *P*-value**
pH1N1 GMFR	-0.220	0.14
H3N2 GMFR	-0.202	0.20
Flu B GMFR	-0.034	0.81
H1 HA-specific CD4+ fold change	-0.170	0.32
H3 HA-specific CD4+ fold change	0.183	0.29
Flu B HA-specific CD4+ fold change	0.291	0.06

Supplementary figure 2. Correlation between enrichment of interferon signaling genes at baseline and immune responses to LAIV at day 21 after vaccination; related to Figure 2. Spearman's rank correlation coefficients (Rho) between normalized enrichment scores (NES) of the interferon signaling pathway generated using a single sample gene set enrichment analysis (GSEA) and antibody or T-cell responses to LAIV at day 21 (compared to baseline). Antibody response is expressed as the fold rise in the haemagglutinin inhibition (HAI) geometric mean titre after vaccination (geometric mean fold rise=GMFR) and CD4+ T-cell response as the fold change after vaccination in the percentage of CD4+ T-cells expressing interferon gamma (IFNg) following stimulation with strain-specific haemagglutinin (HA) peptides.

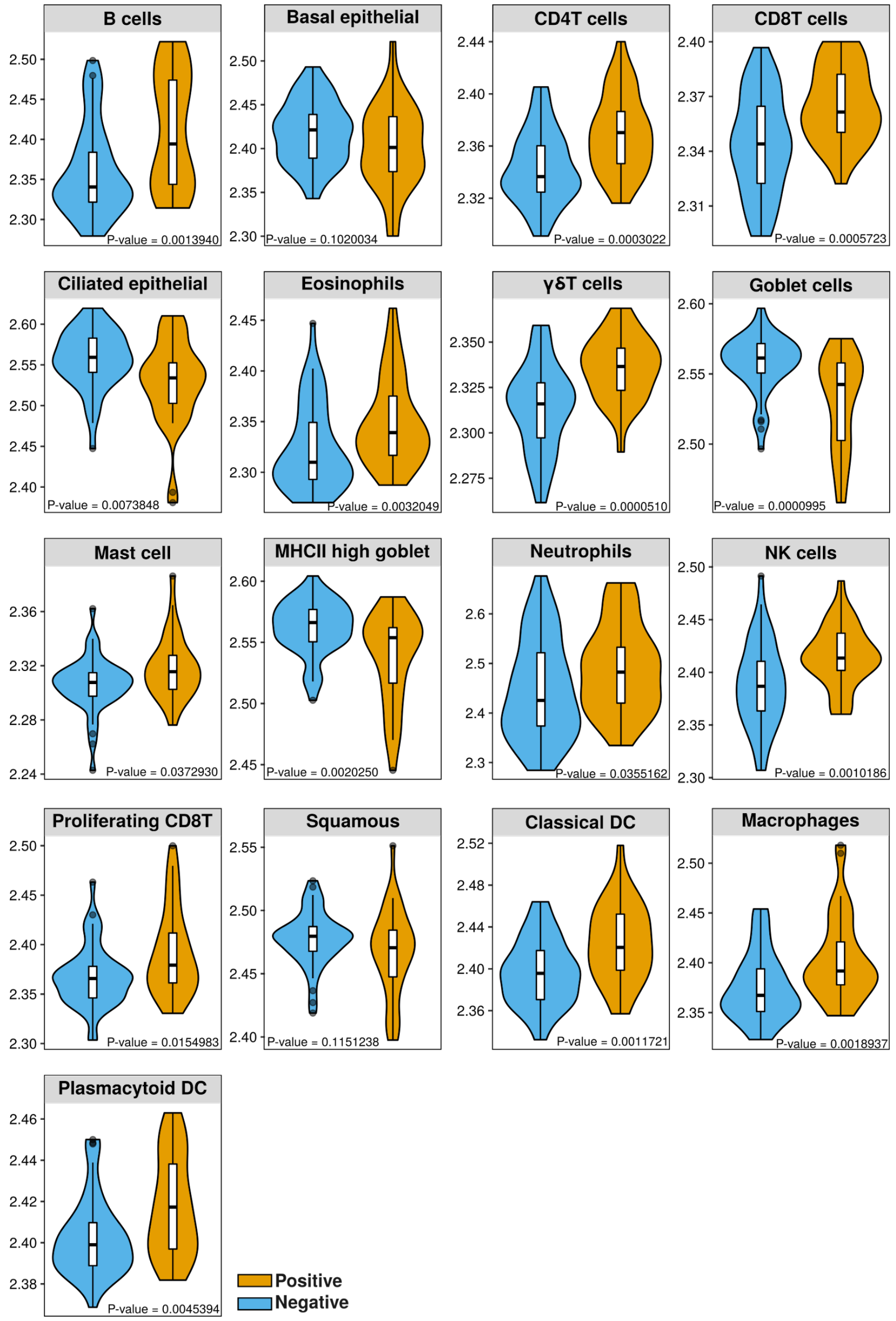

Supplementary figure 3. Comparison of cell-specific gene expression scores between children with and without asymptomatic viruses detected at baseline; related to Figure 3. Sum of normalized expression values from 50 genes defining each cell type[1] for individuals with (positive) and without (negative) asymptomatic virus at baseline compared (Mann Whitney U test).

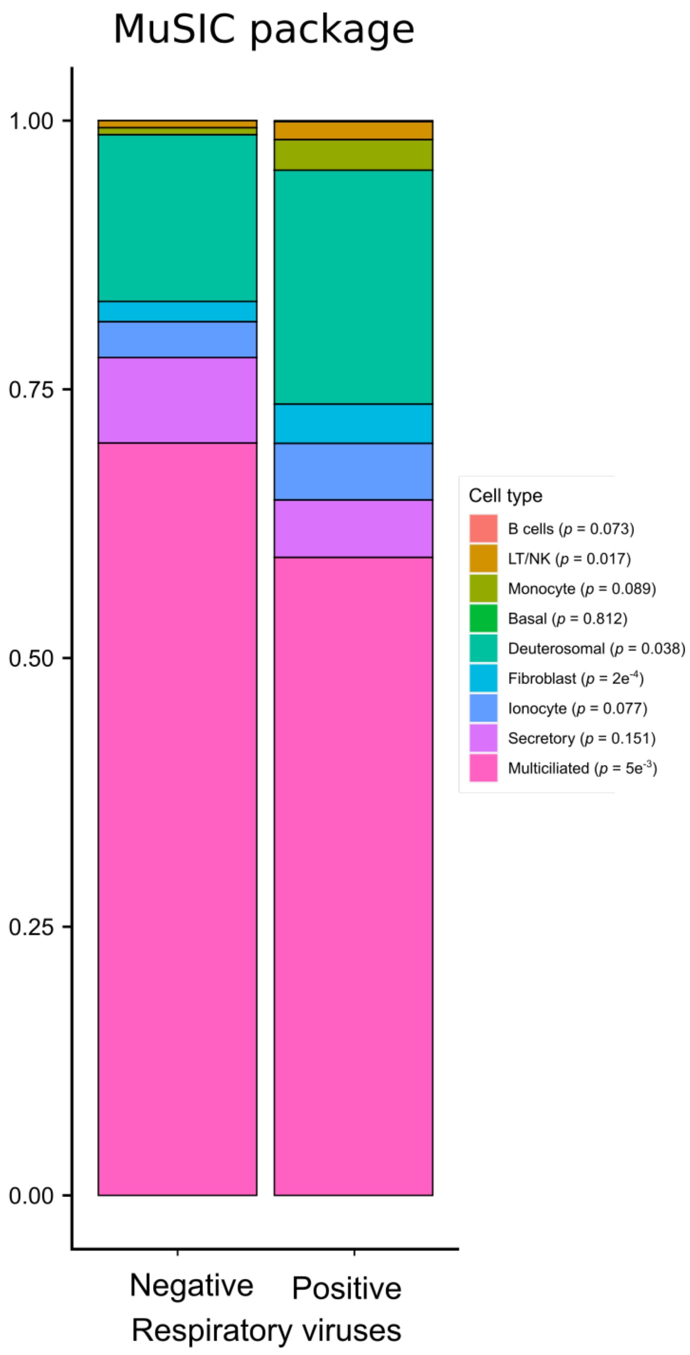

Supplementary figure 4. Comparison of proportion of each cell-type in deconvoluted nasopharyngeal bulk-RNAseq signature between children with and without asymptomatic viruses detected at baseline; related to Figure 3. Proportions of cell-types were calculated using the MuSIC package[2] single-cell atlas data from human healthy airway tissue. [3] P-values are derived from Mann Whitney U test.

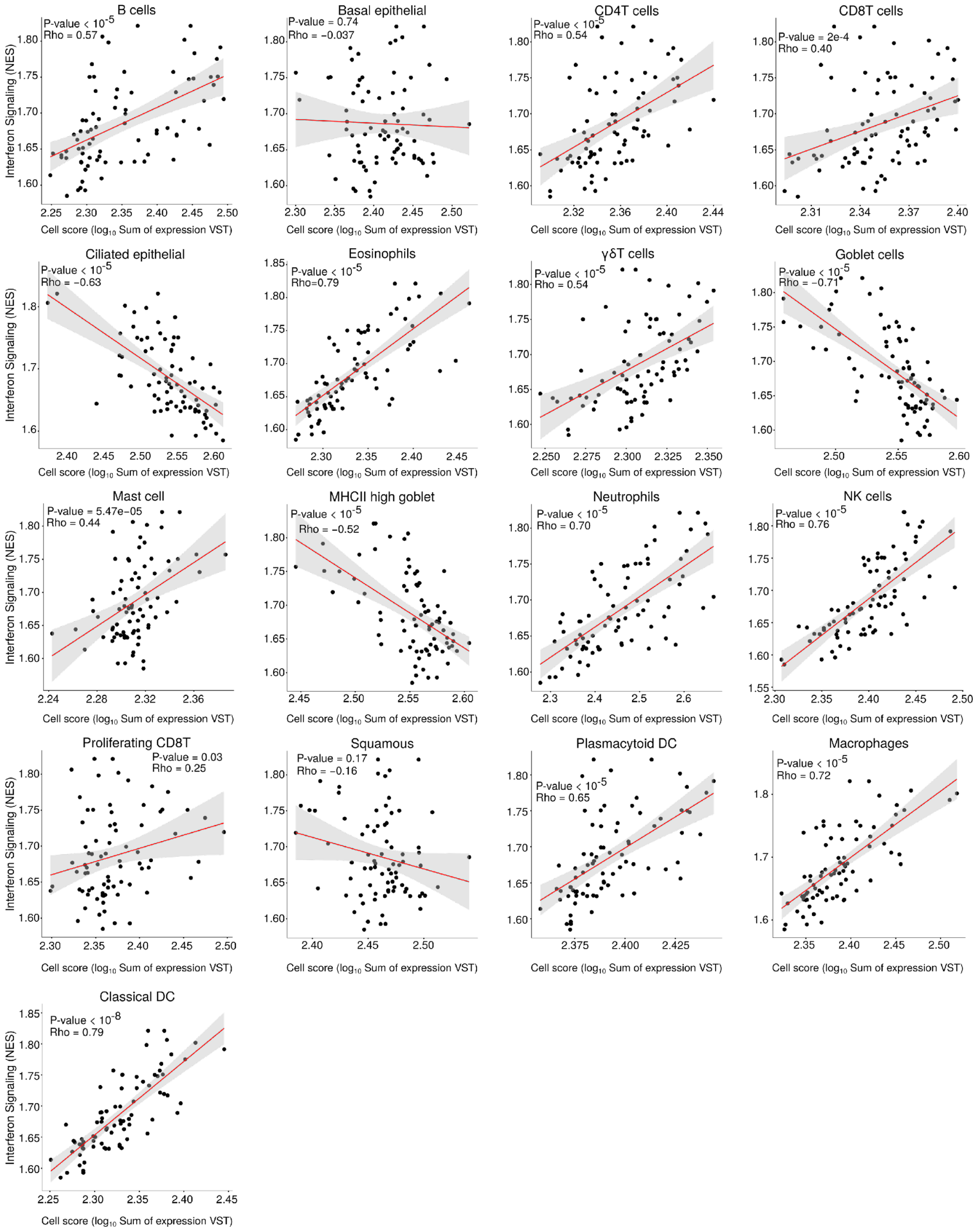

Supplementary figure 5. Correlation between normalized enrichment score (NES) for interferon pathway genes and cell type-specific gene expression signatures at baseline; related to Figure 3. R^2 = Spearman correlation coefficient. Interferon signaling pathway NES derived from single sample gene set enrichment analysis. Cell type-specific score were calculated following exclusion of overlapping genes with those in interferon signaling pathways (Reactome): Classical DCs (7 genes: HLA-DP1, HLADPB1, HLA-DQA1, HLA-DQB1, HLA-DQB2, HLA-DRB5, IRF8); B cells (3 genes: HLA-DQA1, HLA-DQA2, IRF8); Gamma-delta T-

cells (2 genes: IFNG, NCAM1); plasmacytoid DCs (2 genes: IRF4, IRF8); Proliferating CD8 T-cells (1 gene: IFN-G); Squamous cells (1 gene: TRIM2); Neutrophils (1 gene: FCGR1B).

References

1. Cao, Y., Guo, Z., Vangala, P., Donnard, E., Liu, P., McDonel, P., Ordovas-Montanes, J., Shalek, A.K., Finberg, J.P., and Garber, M. (2020). Single-cell analysis of upper airway cells reveals host-viral dynamics in influenza infected adults. bioRxiv. doi.org/10.1101/2020.04.15.042978.
2. Wang, X., Park, J., Susztak, K., Zhang, N.R., and Li, M. (2019). Bulk tissue cell type deconvolution with multi-subject single-cell expression reference. Nat Commun *10*, 380. 10.1038/s41467-018-08023-x.
3. Deprez, M., Zaragosi, L.E., Truchi, M., Becavin, C., Ruiz Garcia, S., Arguel, M.J., Plaisant, M., Magnone, V., Lebrigand, K., Abelanet, S., et al. (2020). A Single-cell Atlas of the Human Healthy Airways. Am J Respir Crit Care Med. 10.1164/rccm.201911-2199OC.
